# Irremediable Psychiatric Suffering in The Context of Medical Assistance in Dying: A Delphi-Study

**DOI:** 10.1177/07067437221087052

**Published:** 2022-03-21

**Authors:** Sisco M.P. van Veen, Natalie Evans, Andrea M. Ruissen, Joris Vandenberghe, Aartjan T.F. Beekman, Guy A.M. Widdershoven

**Affiliations:** 1 Department of Psychiatry, Amsterdam University Medical Center, the Netherlands; 2 Department of Ethics, Law and Humanities, Amsterdam University Medical Center, the Netherlands; 3 113 Suicide prevention, Amsterdam, the Netherlands; 4 Department of Psychiatry, University of Leuven, Belgium

**Keywords:** physician-assisted death, medical assistance in dying, irremediability, ethics, Delphi

## Abstract

**Objective:**

Patients with a psychiatric disorder are eligible to request medical assistance in dying (MAID) in a small but growing number of jurisdictions, including the Netherlands and Belgium. In Canada, MAID for mental illness will become possible in 2023. For this request to be granted, most of these jurisdictions demand that the patient is competent in her request, and that the suffering experienced is unbearable and irremediable. Especially the criterion of irremediability is challenging to establish in patients with psychiatric disorders. The aim of this research is to establish what criteria Dutch and Belgian experts agree to be necessary in characterising irremediable psychiatric suffering (IPS) in the context of MAID.

**Methods:**

A two-round Delphi procedure among psychiatrists with relevant experience.

**Results:**

Thirteen consensus criteria were established: five diagnostic and eight treatment-related criteria. Diagnostically, the participants deem a narrative description and attention to contextual and systemic factors necessary. Also, a mandatory second opinion is required. The criteria concerning treatment show that extensive biopsychosocial treatment is needed, and the suffering must be present for several years. Finally, in the case of refusal, the participants agree that there are limits to the number of diagnostic procedures or treatments a patient must undergo.

**Conclusions:**

Consensus was found among a Dutch and Belgian expert group on potential criteria for establishing IPS in the context of MAID. These criteria can be used in clinical decision-making and can inform future procedural demands and research.

## Abrégé

**Objectif:**

Les patients souffrant d’un trouble psychiatrique sont admissibles à demander l’aide médicale à mourir (AMM) dans un nombre modeste mais croissant d’administrations, notamment les Pays-Bas et la Belgique. Au Canada l’AMM pour la maladie mentale deviendra possible en 2023. Pour que cette demande soit accordée, la plupart des administrations exigent que la patiente soit compétente pour faire sa demande, et que la souffrance éprouvée est insupportable et irrémédiable. Le critère d’irrémédiabilité est spécialement difficile à établir chez les patients souffrant de troubles psychiatriques. La présente recherche vise à établir quels critères sont nécessaires selon une entente des experts néerlandais et belges pour caractériser la souffrance psychiatrique irrémédiable (SPI) dans le contexte de l’AMM.

**Méthodes:**

Une procédure Delphi en deux étapes ‘avec comme participants des psychiatres ayant une expérience pertinente’.

**Résultats:**

Treize critères de consensus ont été établis: cinq critères liés au diagnostic et huit au traitement. Sur le plan diagnostique, les participants jugent nécessaires une description narrative et une attention aux facteurs contextuels et systémiques. Aussi une deuxième opinion obligatoire est requise. Les critères concernant le traitement indiquent qu’un traitement biopsychosocial prolongé est nécessaire, et que la souffrance doit être présente depuis plusieurs années. Enfin, dans le cas d’un refus de traitement, les participants conviennent qu’il y a des limites au nombre de procédures diagnostiques ou de traitements qu’un patient peut subir.

**Conclusions:**

Un consensus a été atteint parmi un groupe d’experts néerlandais et belges sur des critères potentiels concernant la SPI dans le contexte de l’AMM. Ces critères peuvent servir à la prise de décision clinique et peuvent éclairer les futures demandes de procédure et la recherche.

## Introduction

Patients with a psychiatric disorder (PPD) are eligible to request medical assistance in dying (MAID) in a small but growing number of jurisdictions, including the Netherlands and Belgium. In Canada, after March 2023, people with a mental illness as their sole underlying medical condition will have access to MAID. For this request to be granted, most of these jurisdictions demand that the patient is competent in her request and has a medical condition that causes irremediable and unbearable suffering.^1,2^

The criterion of irremediability is particularly difficult to establish in psychiatric disorders.^3^ Retrospective casefile studies of Dutch PPD who died through MAID show if experts disagree on a criterion, it often concerns irremediability.^1,4^ A Dutch euthanasia expertise centre recently stated that more clarity on psychiatric irremediability is a priority.^5^ A Canadian expert advisory group has also called for more research on the irremediability of psychiatric suffering.^6^

A recent qualitative study on irremediable psychiatric suffering (IPS) among Dutch psychiatrists with experience in establishing IPS in the context of MAID concluded that consensus criteria are needed to guide current clinical decision-making.^7^ Moreover, jurisdictions debating MAID for PPD in the future may benefit from the availability of potential criteria for establishing IPS, developed from the relatively longer practice experiences in the Netherlands and Belgium. If jurisdictions choose to allow MAID for PPD, necessary criteria for IPS should also be specified when drafting legislation. Finally, researchers can use the criteria of IPS for designing clinical studies on the irremediability of psychiatric suffering and ways to manage this.

In this study, we use a Delphi method to develop expert consensus criteria for IPS in the context of MAID, focusing on the Netherlands and Belgium where MAID has been permitted in psychiatric practice for over 20 years. Therefore, we address the following research question: what are the criteria that Dutch and Belgian experts agree upon for IPS in the context of MAID for PPD?

## Methods

### Ethical Approval, Data-Management, Study Design and Preregistration

Ethical approval for this study was obtained from the Medical Ethical Examination Board of Amsterdam UMC/VUmc under registration number D326. Furthermore, a privacy impact assessment was performed, assuring compliance with European privacy laws. For data management, the survey tool *Survalyzer* was used, quantitative analysis was performed using *SPSS v25.0*, and for qualitative analysis, *Maxqda v18.2.4* was used. Also, the ‘Conducting and REporting of DElphi Studies’ (CREDES) guidelines were followed.^8^ The study protocol was preregistered at the Open Science Framework under project code: qx5hy.

### Inclusion Criteria

In order to be included in the study (1) participants had to have at least five years of clinical experience as a psychiatrist and (2) they had to have experience with assessing PPD requesting MAID. This could mean that they had investigated a persistent MAID request from one of their own patients, that they acted as an independent clinical expert in a MAID procedure, or both. It was not required for them to have actually assisted a patient in dying. No specific exclusion criteria were used.

### Participant Selection

First, the project group was formed, consisting of the authors of this article, who are Dutch and Belgian experts in MAID in PPD with backgrounds in psychiatry and ethics and hold different views on MAID for PPD. Through criterion recruitment from the clinical and scientific network of the project group, participants were selected in the Netherlands and Belgium. Diverse perspectives were aimed for by purposely inviting psychiatrists who are known proponents, opponents, or hold a moderate stance on MAID for PPD. Participants in the study were also asked to recommend other experts that met the inclusion criteria *(snowballing)*. An information letter was sent describing that participation will not yield direct benefits and that the main burden is the time investment. Informed consent was obtained from all participants, and everyone gave permission to be acknowledged for their efforts in the final publication, adding to the transparency of the study. Participants were sent an email with a personal link to the online survey. At the beginning of the study, all round 1 participants were explicitly asked to participate in all subsequent rounds, and during both rounds, two reminders were sent.

### Survey Design and Data Analysis

The round 1 survey was developed during project group meetings using insights from a systematic review and a qualitative interview study among psychiatrists, both on the topic of IPS.^3,7^ The criteria were subdivided into three categories: diagnostic criteria, treatment criteria and treatment refusal criteria. First, the participants were asked to give their own definition of IPS. Next, they were asked their opinions on 20 criteria using a 5-point Likert scale (strongly disagree, disagree, neutral, agree, strongly agree). The participants were encouraged to provide arguments for their ratings in an open comment section. Relevant socio-demographic and professional characteristics were also collected (Table 1). Finally, participants were asked whether they would perform MAID for PPD themselves. The survey was validated in a pilot phase, during which three senior psychiatry residents from the Netherlands and Belgium filled out the survey in the presence of the corresponding author using the ‘think aloud’ approach.^9^

**Table 1.** Respondent Characteristics.

	Round 1 (*n* = 53)	Round 2 (*n* = 47)
Age, mean (SD)	54 (10)	54 (9.9)
Female respondents (%)	25 (47)	23 (49)
Religion (%)		
Non-religious	42 (89)	37 (79)
Christian	8 (15)	7 (15)
Other religion^a^	1 (2)	1 (2)
No answer	2 (4)	2 (4)
Country of occupation^b^ (%)		
Netherlands	40 (75)	36 (77)
Belgium	11 (21)	9 (19)
Netherlands and Belgium	2 (4)	2 (4)
Years of clinical experience^c^ (SD)	22.3 (9.8)	22.4 (9.7)
Primary workplace (%)		
1^st^ tier psychiatric practice	1 (2)	1 (2)
2^nd^ tier psychiatric care facility	18 (34)	16 (34)
General hospital	8 (15)	7 (15)
Forensic psychiatric care facility	1 (2)	1 (2)
3^rd^ tier psychiatric care facility	10 (19)	9 (19)
University Hospital	8 (15)	6 (13)
Expertise Centre Euthanasia	5 (9)	5 (11)
(Independent) Euthanasia Consultant	2 (4)	2 (4)
Sub-specialization^d^ (%)		
Child and adolescent psychiatry	5 (9)	5 (11)
Adult psychiatry	45 (85)	39 (83)
Elderly psychiatry	10 (19)	10 (21)
Clinical expertise^d^ (%)		
Anxiety disorders	12 (23)	10 (21)
Depressive mood disorders	17 (32)	15 (32)
Bipolar disorders	17 (32)	16 (34)
Trauma- and stressor-related disorders	8 (15)	6 (13)
Neurobiological development disorders	9 (17)	7 (15)
Neurodegenerative disorders	10 (19)	9 (19)
Obsessive-compulsive disorders	11 (21)	9 (19)
Personality disorders	18 (34)	16 (34)
Schizophrenia and related psychotic disorders	20 (38)	16 (34)
Somatic symptom disorders	8 (15)	6 (13)
Eating disorders	5 (9)	4 (9)
Other psychiatric disorders	15 (28)	13 (28)
Experience with medical assistance in dying (MAID) for psychiatric suffering^d^ (%)		
Received a MAID-request from a patient under their care	48 (91)	42 (89)
Performed an independent consultation	43 (81)	39 (83)
Have performed MAID themselves	12 (23)	11 (23)
Views on performing MAID for psychiatric suffering (%)		
Would consider performing MAID	29 (55)	28 (60)
Would not consider performing MAID	12 (23)	9 (19)
Unsure about performing MAID	11 (21)	9 (19)
I would rather not answer	1 (2)	1 (2)

MAID = medical assistance in dying.

^a^ This option was selected from a list containing all major religions.

^b^ Since it is possible to life in one country and work in the other, we focused on the country of occupation, rather than nationality. It is possible for clinicians to work in both countries.

^c^ Counted from the moment they became a psychiatrist.

^d^ Categories are not mutually exclusive.

After round 1, the open definition and accompanying comments for each criterion were coded and categorized using thematic analysis, paying particular attention to indications that the participant had misunderstood any elements of the criteria or desired more details.^10^ The Likert scales were analysed using basic descriptive statistics. Consensus was defined as 70% of the experts agreeing/disagreeing or strongly agreeing/disagreeing with a statement (i.e., the top or bottom two options on the five-point Likert scale).^11^ The round 1 results were discussed in two project group meetings and summarized in a feedback report (supplement 1). The open definitions served as inspiration for additional criteria for round 2. When the comments showed that criteria were misunderstood or valuable suggestions were given for wording changes, the criteria were modified and included in round 2 accompanied by a summary of the comments. If the comments lacked relevant arguments and no substantial changes were suggested, the consensus or dissensus about the criterion was accepted. The round 2 survey was piloted again on one of the senior psychiatric residents using the ‘think aloud’ approach.

The results of round 2 were discussed in a project group meeting and summarized in a feedback report (supplement 2). Using the same standards as round 1 it was concluded that the wording was sufficiently clear for all criteria, arguments for agreeing or disagreeing were similar to round 1 and became repetitive, and no substantial new viewpoints were introduced by participants, indicating response stability.^12^ Therefore, the outcomes of round 2 criteria were accepted and no third round was performed.

## Results

### Participants

Sixty-seven psychiatrists, meeting the inclusion criteria, responded to an invitation to participate and were sent the survey; 53 psychiatrists completed the first round (79%), of these 47 completed round 2 (89% of those responding to round 1). Demographic and professional characteristics of participants are shown in Table 1. Of the initial 53 participants, the mean age was 54 and 47% were female. Of these participants, 75% worked in the Netherlands, 21% in Belgium and 4% in both countries; 91% had received a MAID request from one or more of their own patients, 81% had performed an independent consultation and 23% had actually performed MAID due to psychiatric suffering.

### Criteria

After two Delphi rounds, consensus was reached for 13 criteria, which can be subdivided into five diagnostic and eight treatment-related criteria (Table 2). Below we summarize the iterative process resulting in the consensus criteria.

**Table 2.** Consensus Criteria for Irremediable Psychiatric Suffering in the Context of Physician-Assisted Death.

Diagnostic criteria
A. When establishing irremediable psychiatric suffering:
1. A psychiatric diagnosis, as described in the DSM-5, should be established according to applicable guidelines.
2. In addition to the diagnosis according to the DSM-5, a narrative account must be given that includes aetiology and pathogenesis.
3. In addition to the diagnosis according to the DSM-5, it should be standard practice to verify whether there are contextual or systemic factors that cause or maintain the psychiatric complaints.
B. During the MAID assessment, the diagnosis must be independently confirmed by at least two psychiatrists.
C. There are limits to the number of new diagnostic procedures a patient must undertake before it can be said that the psychiatric suffering is irremediable. *For example: a patient or psychiatrist may refrain from further diagnostic procedures on reasonable grounds, such as a long history of illness and treatment.*
Treatment criteria
D. If side effects allowed, the indicated drug treatments should have been adequately performed without leading to a significant reduction in suffering.
E. If side effects allowed and if indicated, electroconvulsive therapy (ECT) should have been attempted for a sufficient length of time without leading to a significant reduction in suffering.
F. Psychotherapeutic treatments indicated by the applicable guideline must have been attempted without leading to a significant reduction in suffering.
G. If there are indications that entering into a repeated psychotherapeutic trajectory is meaningful, this must be offered before irremediable psychiatric suffering can be established. *For example: because conditions were sub-optimal in previous therapy.*
H. At least one recovery-oriented treatment must have been attempted without leading to a significant reduction in suffering.^a^
I. If necessary, substantial efforts should have been made to improve the patient's social situation without leading to a significant reduction in suffering.
J. Because all reasonable treatments must be tried, the psychiatric suffering must have been present for several years before irremediable psychiatric suffering can be established.
K. There are limits to the number of treatments a patient must undergo before psychiatric suffering can be considered irremediable. *For example, a patient or psychiatrist may refrain from further treatment on reasonable grounds, such as a long history of illness and treatment or the prospect of serious side effects.*

^a^ When describing recovery, the corresponding Dutch guideline, used the definition of Anthony (1993), which sees recovery as “an individual process aimed at rediscovering one's personal identity and taking back control of one's life.”.

#### Open Definition

At the beginning of round 1, the participants were asked to give an open definition of IPS in the context of MAID; 52 participants gave detailed definitions. Through thematic analysis, recurrent themes were identified which are summarized below. A full report of the qualitative analysis can be found in supplement 1.

Most participants’ definitions specified that a psychiatric disorder should cause suffering which is *persistent, long lasting, chronic or constant*. Also, almost all definitions contained a criterion that the prognosis should be poor, or as one participant defines it:

[IPS is] severe suffering that stems from a psychiatric disorder and cannot be alleviated by any available treatment options.—P16, Dutch, age 50, has experience with MAID as an independent expert.

Various participants added that extensive treatment must have been tried and failed. Several emphasized the importance of ‘finishing the treatment-protocol’ or ‘trying all evidence-based treatments’ without relief of suffering. Others explicated that only reasonable treatment options can be demanded from the patient. One participant captured both of these perspectives, stating:

Subjectively severe suffering linked to one or more psychiatric diagnoses for which the various treatment options advised by guidelines and accepted within reasonable limits by the patient have been exhausted.—P31, Belgian, age 60, has experience with MAID as an independent expert.

The themes ‘persistence of suffering’, ‘poor prognosis’ and ‘failed treatment’ led to two new criteria for round 2 (see section on *round 2 criteria* below).

#### Round 1 Criteria

Round 1 was subdivided into diagnostic criteria, treatment-related criteria and treatment refusal criteria (Table 3). Eight of 20 criteria reached consensus, three of which were diagnostic criteria (Table 2: A1, A3 and B), five were treatment criteria (Table 2: D, E, F, H & I), none of the treatment refusal criteria reached consensus.

**Table 3.** Likert-Scale Scores of Round 1 Criteria.

Diagnostic criteria	Disagree/strongly disagree	Agree/strongly agree	Action after analysing the comments
A psychiatric diagnosis, as described in the DSM-5, should be established according to applicable guidelines.	13%	83%	Accepted
During the MAID-procedure the diagnosis must be independently confirmed by at least two psychiatrists.	8%	83%	Accepted
In addition to the descriptive diagnostics according to the DSM-5, it should be standard practice to verify whether there are contextual or systemic factors that cause or maintain the psychiatric complaints.	0%	100%	Accepted
Broad psycho-diagnostic testing, including personality testing, should be the standard, unless the psychiatrist provides clear reasons why it is not necessary.	36%	41%	Rephrased without the words ‘broad’ and ‘standard’
In addition to the descriptive diagnostics according to the DSM-5, a formulation must be drawn up for each patient based on a psychotherapeutic model relevant to the disorder.	30%	43%	Changed ‘a psycho-therapeutic model’ to ‘a narrative account’
Treatment criteria	Disagree or strongly disagree	Agree or strongly agree	Action after analysing the comments
If side effects allow it, the indicated drug treatments should be adequately performed without leading to a significant reduction in suffering.	0%	98%	Accepted
If side effects allow it and if indicated, ECT should have been attempted for a sufficient length of time without leading to a significant reduction in suffering.	9%	79%	Accepted
Psychotherapeutic treatments indicated by the applicable guideline must have been attempted without leading to a significant reduction in suffering.	2%	92%	Accepted
If necessary, substantial efforts should be made to improve the patient's social situation without leading to a significant reduction in suffering.	0%	92%	Accepted
At least one recovery-oriented treatment must have been attempted without this leading to a significant reduction in suffering.	8%	72%	Accepted
When indicated, psychosurgical treatment (such as DBS) must have been attempted without significantly reducing suffering.	39%	32%	Changed ‘attempted’ to ‘offered’
If indicated, at least one acceptance-oriented psychotherapy must have been attempted without leading to a significant reduction in suffering before it can be considered irremediable.	9%	60%	Changed ‘before it can be considered irremediable’ to ‘before IPS can be established’
Indicated psychotherapeutic treatments that were ineffective in the past, should be repeated without leading to a significant reduction in suffering.	51%	17%	Changed ‘demanded’ to ‘offered’ and added that therapy should only be repeated ‘if there are indications that this is meaningful’
Treatment refusal criteria	Disagree or strongly disagree	Agree or strongly agree	Action after analysing the comments
If a patient does not want to participate in the diagnostic process, there can be no irremediable psychiatric suffering.	26%	49%	Rephrased to ‘there are *limits* to the number of diagnostic procedures a patient must undertake’
When a patient refuses the above-mentioned drug treatments, the suffering is not irremediable.	23%	53%	Merged all criteria into one more generic criterion about treatment and changed the wording to ‘there should be limits to the number of treatments a patient can be asked to undergo’
When a patient refuses the above-mentioned ECT, the suffering is not irremediable.	34%	36%	
When a patient refuses the above-mentioned psycho-surgical treatment, the suffering is not irremediable.	60%	21%	
When a patient refuses the above-mentioned psychotherapy, the suffering is not irremediable.	17%	57%	
When a patient refuses the above-mentioned acceptance-oriented psychotherapy, the suffering is not irremediable.	23%	47%	
When a patient refuses the above-mentioned repetition of psychotherapy, the suffering is not irremediable.	47%	11%	

DSM-5 = diagnostic statistical manual, fifth edition; MAID = medical assistance in dying; ECT = electroconvulsion therapy; IPS = irremediable psychiatric suffering; DBS = deep brain stimulation.

Three of five diagnostic criteria reached consensus in the first round (Table 3). The accompanying comments indicated these criteria were sufficiently clear and that no substantial new viewpoints were introduced in the comments. These were not, therefore, repeated in round 2. Two diagnostic criteria that did not reach consensus were included in round 2 after rephrasing guided by the comments (Tables 3 and 4).

**Table 4.** Likert-Scale Scores of Round 2 Criteria.

Diagnostic criteria	Disagree or strongly disagree	Agree or strongly agree	Action after analysing the comments
Structured psycho-diagnostic testing, including personality testing when relevant, should be performed, unless the psychiatrist provides clear reasons why it is not necessary.	32%	55%	Accepted
When establishing irremediable psychiatric suffering a narrative account must be given, that includes aetiology and pathogenesis, in addition to the classification according to the DSM-5.	2%	91%	Accepted
There are limits to the number of new diagnostic procedures a patient must undertake before it can be said that the psychiatric suffering is irremediable. *For example: a patient or psychiatrist may refrain from further diagnostic procedures on reasonable grounds, such as a long history of illness and treatment.*	6%	81%	Accepted
Treatment criteria	Disagree or strongly disagree	Agree or strongly agree	Action after analysing the comments
Because it is often difficult to establish a reliable prognosis, the judgment about non-remediable psychiatric suffering must be based on the failure of treatment in the past.	11%	66%	Accepted
Because all reasonable treatments must be tried, the psychiatric suffering must be present for several years before irremediable psychiatric suffering can be established.	15%	81%	Accepted
If indicated, psychosurgery (such as DBS) must be discussed and offered to the patient before irremediable psychiatric suffering can be established.	28%	62%	Accepted
If indicated, at least one acceptance-oriented psychotherapy must have been attempted without leading to a significant reduction in suffering before irremediable psychiatric suffering can be established.	13%	66%	Accepted
If there are indications that entering into a repeated psychotherapeutic trajectory is meaningful, this must be offered before irremediable psychiatric suffering can be established. *For example: because conditions were sub-optimal in previous therapy.*	4%	70%	Accepted
There are limits to the number of treatments a patient must undergo before it can be referred to as irremediable psychiatric suffering. *For example, patient or psychiatrist may refrain from further treatment on reasonable grounds, such as a long history of illness and treatment and/or the prospect of serious side effects.*	11%	81%	Accepted

DSM-5 = diagnostic statistical manual, fifth edition; DBS = deep brain stimulation.

Of the eight initial treatment criteria, five reached consensus in the first round (Table 3). From the comments, it was clear that all criteria were understood and these were not repeated in round 2. Three other criteria did not reach consensus and were adapted based on participants’ comments and repeated in round 2 (Tables 3 and 4).

None of the treatment refusal criteria reached consensus. This appeared to be due to the formulation of the criteria: many participants commented that it is certainly possible that the suffering is irremediable when the patient does not cooperate, but that the irremediability cannot be established in this case. The criteria were reformulated in round 2 (Table 4).

#### Round 2 Criteria

The second round contained nine criteria (Table 4). The open definition in round 1 inspired two new criteria in round 2. Of these, one reached consensus (Table 2: criterion J). Two diagnostic criteria reached consensus (Table 2: criteria A2 and C). Out of three criteria concerning treatment, two reached consensus (Table 2: criteria G and K). The comments showed that all criteria were well understood and therefore both the dissensus and the consensus that was found were regarded as valid and no new round was started.

## Discussion

Through a modified Delphi method, 13 expert consensus criteria for IPS in the context of MAID were identified, regarding diagnosis and treatment.

### Diagnostic Criteria

Participants considered a DSM classification necessary when determining IPS, but even more, participants considered the presence of a narrative description and attention to psychosocial factors important. Both the psychosocial context in which the complaints have emerged and endured and their influences on the suffering, have to be taken into consideration. These criteria are in line with clinical guidelines describing best practice in psychiatric evaluation.^13,14^ The criteria imply that a certain degree of individual interpretation will always be part of the decision-making process concerning IPS in the context of MAID. Further deliberation should focus on what levels of individual interpretation are justifiable.^15^

All participants agreed that a mandatory second opinion should be a criterion of IPS in the context of MAID. This endorses the current due diligence procedures in the Netherlands and Belgium.^16–18^ Furthermore, a second opinion can mitigate the risk of interpretative differences. Canadian policymakers should take this insight into account when developing the due diligence procedures for psychiatric MAID.

The participants also agree that, although there must be evidence of a substantial clinical history (described in detail below), there should be limits to the number of diagnostic procedures a patient has to undergo before IPS can be established. Especially when this patient has a long history of illness and treatment. This criterion suggests that if the patient refuses certain diagnostic procedures, it does not automatically mean that IPS cannot be established. It may be justified to only demand additional diagnostic procedures, such as neuropsychological or personality testing, when there is a reasonable chance that this will lead to new treatment options with a high probability of reducing suffering.

### Treatment Criteria

The participants agree that substantial treatments have to have failed in reducing suffering before IPS can be established. This is in line with earlier studies.^3,4,19^

The participants also agree that, because all reasonable treatments must be tried, the suffering should be present for several years. This criterion reflects the notion that the persistence of suffering is not only time-dependent but also treatment dependent.^20^ Implementation of this criterion as a due diligence requirement might provide clarity for patients and regulators about the high threshold of irremediability in the context of MAID for PPD. It may also be relevant for distinguishing MAID requests from impulsive suicidality.^21^

The results also show that experts take both biological and psychological treatments, and social interventions, into account when establishing IPS. This is in line with the biopsychosocial model of psychiatric suffering and treatment, which was introduced by George Engel in 1977, and is still highly influential in contemporary psychiatry.^22^ In the context of MAID, it can serve as a helpful framework to assess individual treatment criteria.

Regarding the biological treatments, there is consensus that medication and ECT should have been tried, but not psychosurgery. Based on the comments, two main reasons for dissensus emerge: participants find psychosurgery too experimental, too invasive, or both. This shows that effectiveness and proportionality should be taken into account when deciding on treatment criteria.

Participants also agree that appropriate psychotherapeutic treatments must have failed before IPS can be established. Psychiatrists especially supported repetition of psychotherapy if there are indications that earlier therapy was ‘performed inadequately’. This criterion should be used with caution, as it is difficult to reliably evaluate the quality of earlier psychotherapy, and knowledge about the efficacy of repeated psychotherapy for therapy-resistant psychiatric complaints is lacking.^7,23^ There was no consensus regarding the criterion that acceptance-based therapy should be attempted. This is at odds with the suggestion that working towards acceptance of suffering can be seen as a subsidiary option to psychiatric MAID.^3^

The participants consider social interventions, including recovery-oriented approaches, important in the context of MAID. Also, if necessary, substantial efforts should be made to improve the patient's social situation. This may be read as support for the often used argument against MAID stating that when a patient with a psychiatric disorder wants to die, we should improve their situation, not offer MAID.^24^ But the criterion also implies that when there is no need for improving social support or if proper support does not reduce suffering, IPS may still be established and MAID may still be justified.

Finally, there was consensus that there should be limits to the number of treatments a patient has to undergo before IPS can be established. This allows for treatment refusal, which is an important theme in the debate about IPS in the context of MAID. In the Netherlands, the law requires that patient and physician make a shared decision about treatment refusal. In Canada, however, this decision is currently left more to the patient, but it is as of yet unclear how this will change when MAID for psychiatric illness becomes possible in 2023.^3^ However, as none of the specific treatment refusal criteria reached consensus, no conclusions can be drawn regarding specific psychiatric treatments the patient should undergo before IPS can be established in the context of MAID. The current criterion, that only states that there should be *limits,* leaves room for interpretive differences between psychiatrists, patients and other stakeholders. More details on the development of this criterion can be found in supplement 1, pages 22–27. This can be seen as an argument for a diligent assessment of IPS, which requires experience and expertise of participants, as well as joint deliberation, in order to apply the criterion to an individual case. This is not only true for the criterion on treatment refusal but also holds for the criteria in general.

### Strengths and Weaknesses

A strength of this study is that we were able to access a substantial group of psychiatrists with ample experience in assessing MAID requests, and representing different views on psychiatric MAID.

A limitation of a consensus-building Delphi survey is that the structure of the questionnaire is determined by the researchers, and participants’ comments are interpreted by the project group, limiting the influence of the participants. Moreover, no widely accepted benchmark exists within the research community of what constitutes an adequate level of consensus. Also, because we had to change the criteria in between rounds we were not able to calculate response-stability for any of our questions, which could have been a valuable addition to the current qualitative analysis of response stability.^12^ Finally, we did not include relatively new treatments which were less widely used in the Netherlands and Belgium at the time of the study, such as transcranial magnetic stimulation or psychedelic treatments, as potential criteria.

### Recommendations for Practice and Research

We recommend implementing these consensus criteria in the due diligence procedures in the Netherlands and Belgium, in order to contribute to more uniformity and fewer fundamental disagreements when assessing the irremediability of psychiatric suffering in the context of MAID. The potential criteria identified here should be seen as necessary, but not sufficient, and have to be applied with due expertise. Also, more research exploring patient views and the predictive ability of these criteria are needed to make more robust claims about IPS.

On a wider scale, we recommend that other jurisdictions which allow MAID for PPD or are currently discussing options for doing so, will consider the importance of specifying minimum and necessary criteria for the establishment or IPS. We hope that the criteria agreed on by Dutch and Belgian experts can be used to inform the development of guidance in other jurisdictions. We recommend that this study is replicated in other countries to see whether similar criteria are agreed upon.

Although the criteria are specifically established for patients requesting MAID, they can serve as inspiration for examining psychiatric irremediability in general. Psychiatry is beginning to understand how to rationally deploy sequential treatment modalities, but when to stop treatment is rarely discussed. This is problematic, because continuing to treat patients if no effect is to be expected could be harmful, especially when coercion and burdensome procedures are used. By reliably establishing that suffering is irremediable, we may recognize further curative treatment as futile and shift to palliative approaches.^25,26^

## Supplemental Material

sj-pdf-1-cpa-10.1177_07067437221087052 - Supplemental material for Irremediable Psychiatric Suffering in The Context of Medical Assistance in Dying: A Delphi-Study Click here for additional data file. Supplemental material, sj-pdf-1-cpa-10.1177_07067437221087052 for Irremediable Psychiatric Suffering in The Context of Medical Assistance in Dying: A Delphi-Study by Sisco M.P. van Veen, Natalie Evans, Andrea M. Ruissen, Joris Vandenberghe, Aartjan T.F. Beekman and Guy A.M. Widdershoven in The Canadian Journal of Psychiatry

sj-pdf-2-cpa-10.1177_07067437221087052 - Supplemental material for Irremediable Psychiatric Suffering in The Context of Medical Assistance in Dying: A Delphi-Study Click here for additional data file. Supplemental material, sj-pdf-2-cpa-10.1177_07067437221087052 for Irremediable Psychiatric Suffering in The Context of Medical Assistance in Dying: A Delphi-Study by Sisco M.P. van Veen, Natalie Evans, Andrea M. Ruissen, Joris Vandenberghe, Aartjan T.F. Beekman and Guy A.M. Widdershoven in The Canadian Journal of Psychiatry
